# Factors affecting depression among married adults: a gender-based household cross-sectional study

**DOI:** 10.1186/s12889-023-16979-9

**Published:** 2023-10-24

**Authors:** Md. Abdul Wadood, Md. Rezaul Karim, Sheikh Md. Abu Hena Mostafa Alim, Md. Masud Rana, Md. Golam Hossain

**Affiliations:** 1 Health Research Group, Department of Statistics, University of Rajshahi, Rajshahi, Bangladesh; 2 Department of Biochemistry and Molecular Biology, University of Rajshahi, Rajshahi, 6205 Bangladesh; 3 Department of Psychiatry, Rajshahi Medical College, Rajshahi, 6100 Bangladesh

**Keywords:** Depression, Married adults, Gender, Logistic regression model, Bangladesh

## Abstract

**Background:**

Depression is a common mental health problem all over the world including Bangladesh. World Health Organization included it in the Mental Health Gap Action Programme as one of its priority conditions. Research on this issue is scanty in Bangladesh. Therefore, we designed to a gender-based household study on associated factors of depression among married adults in Rajshahi City of Bangladesh.

**Methods:**

We conducted this household cross-sectional study from August 01 to October 31, 2019. A total of 708 married adults currently living together in Rajshahi City were recruited for this study. We applied a multi-stage random sampling technique for selecting samples and used a semi-structured questionnaire to collect necessary information from them. The Patient Health Questionnaire-9 was used for measuring depression and frequency distribution and binary logistic regression model were used for data analysis.

**Results:**

The prevalence of depression (moderate to severe) was 14.4% (95% CI: 11.9–16.9) among married adults, and females (21.2%, 95% CI: 17.2–25.4) suffered more than males (7.6%, 95% CI: 4.8–10.5). A multiple binary logistic regression model established four main factors of depression among married females: (i) multiple marriage [AOR = 19.982; 95% CI: 10.081–39.610; p < 0.01]; (ii) poor relationship with spouse [AOR = 2.175; 95%CI: 1.068–4.428; p < 0.05]; (iii) chronic medical comorbidity [AOR = 1.876; 95%CI: 1.009–2.626; p < 0.05]; and (iv) 7–12 years duration of conjugal life [AOR = 2.091, 1.009–4.334; p < 0.05]. Two main factors of depression among married males were (i) multiple marriage [AOR = 24.605; 95% CI: 20.228–40.402; p < 0.01] and hard work [AOR = 4.358; 95%CI: 1.109–7.132; p < 0.05].

**Conclusion:**

The prevalence of depression was significantly high among the study population, and females were the most vulnerable group. The concerned authorities and stakeholders should take appropriate measures to manage the problem with special focus on the risk factors and the vulnerable groups.

## Introduction

Depressive disorder is a very common mental health problem all over the world [1, 2]. It is manifested by low mood, loss of pleasure or interest in daily activities, sleep disturbance, reduced appetite, fatigue, poor concentration and feeling of worthlessness [3]. It often lasts longer reducing the person’s quality of life [4, 5], and when severe, also causes functional impairment [6]. Furthermore, it may lead the person to attempt and/or commit suicide [7, 8] and increases the risk of mortality [9]. The World Health Organization (WHO) included it in the Mental Health Gap Action Programme as one of its priority conditions [10]. Asia recorded the highest rate of prevalence of depressive disorder (43.0%) compared to Europe (38.0%), Middle East (28.0%), North America (26.0%), and Latin America (21.0%) [11]. In India, the overall prevalence of depression among urban population was 15.1% and in rural area 35.5% [12, 13]. Another Indian study reported 8.9% prevalence rate of depression among elderly population from both urban and rural settings [14]. A systematic review showed that the overall mean prevalence of depression among community samples was 33.62% in Pakistan [15]. A WHO survey reported 47.7%, 40.4%, 40.3%, and 11.4% prevalence rate of depression in Bangladesh, Nepal, India and Sri Lanka respectively [16].

Depressive disorder is a common public health problem in Bangladesh. According to the National Mental Health Survey 2019, the overall prevalence of depressive disorder is 6.7% among the general population in Bangladesh [17]. A study revealed that the prevalence of moderate to severe depression was 69.5% among university students [18]. However, a similar study found 28.7% of university students suffering from depression [19]. Another study got 39.1% of medical students having different levels of depression [20]. About 59% of the indigenous people suffered from depression in Bangladesh [21]. A nationwide survey showed that the overall prevalence of depression was significantly high among adolescents (mild 17.9%, moderate 5.4%, moderately severe 1.1% and severe 0.1%) [22], another study found moderate to severe depression among 26.5% of school going adolescents [23]. A total of 20% of women of reproductive age had major depression [24]. In a study among mothers of school-going children, 15.4% mild, 22.2% moderate and 20.1% severe level of depression were recorded [25]. Among older people, the prevalence rate of depression ranged from 36.9 to 55.5% in Bangladesh [26–28]. About 26.5% of low-income women and 23.5% of female garment workers were screened positive for depression [29]. In these studies, age, gender, socioeconomic status, education level, residence, marital status, stress, traumatic events and co-morbidities were identified to have correlation to depression. However, to the best of our knowledge, gender-based study of depression among married adults is not available yet in Bangladesh. Therefore, we designed to conduct a gender-based household study of depression among married adults in Rajshahi City of Bangladesh.

(i) Our research questions were: What is the prevalence of depression among married adults in Rajshahi city of Bangladesh?
(ii) What are the risk factors of depression among married adults in Rajshahi city of Bangladesh?
(iii) What are the gender-based differences between the risk factors of depression among married adults in Rajshahi city of Bangladesh?

## Methods

### Study design and sampling

This was a household cross-sectional study. The married adults currently living together with their spouses in the households situated in Rajshahi City Corporation (RCC) area were considered as the study population. Since the number of households was known (99,222) [30], the required number of samples for the present study was calculated using the formula, $n = \frac{{{z^2}p(1 - p)}}{{{d^2}}}$ [31], where n = the number of samples, z = 1.96, p = 0.302 (the prevalence of depression among married women was taken from a previous study [32], and d = 0.05 (margin of error). The formula provided that 324 households were the minimum required sample size for the study. However, we decided to select 375 households considering the risk of non-participation of some selected respondents. The targeted number of households was selected using a multistage random sampling technique. RCC is administratively divided into 30 ‘Wards’ and each Ward comprises several neighborhoods known as ‘Muhalla’. In the first stage, we randomly chose three Wards. In the second stage, we selected five Muhallas from each of the chosen Wards by simple random sampling. In the third stage, 25 households were selected from each chosen Muhalla using simple random sampling. Thus, a total number of 375 households (3 × 5 × 25 = 375) were selected for this study, and one couple (two married adults currently living together) from each of the selected households was selected to have 750 married adults for analysis. In the case of more couples in a household, one couple was selected by lottery. All information regarding Wards, Muhallas and households were gathered from RCC and respective Ward Councilor offices.

### Data collection

We conducted the survey during the period of August 01 to October 31, 2019. We used a semi-structured questionnaire for collecting data that included nine questions of the Patient Health Questionnaire (PHQ-9) with other questions and statements for anthropometric, demographic, socio-economic, household, familial and health-related information of the respondents. The first author drafted the questionnaire in English, and sent it to three experts in psychology; and it was finalized incorporating the experts’ comments/suggestions/opinions. The final English questionnaire was then translated into Bangla by the first author so that the respondents understand the questions easily. The draft Bangla-version of the questionnaire was sent to two language experts and a psychologist for revision and editing. The Bangla-version questionnaire was finalized after correcting it based on suggestions of the experts. We trained three teams of interviewers for the survey. One team comprised of one male and one female students of master’s level of the Department of Statistics in Rajshahi University. They went to the selected households, explained the objectives and methods of the study to them and took their written consent for participating in the study. A total of 21 couples declined to give their information and 354 couples (708 married adults) gave their consent and took part in the survey.

### Outcome variable

Depression was the outcome variable of this study that was determined by the PHQ-9. It is a highly sensitive and specific scale for screening depression during last two weeks that was prepared with depression-related nine statements from the 3-page PHQ questionnaire [33]. This is widely-used all over the world including Bangladesh [22, 34, 35]. Each of the PHQ-9 statements has four-point scale: not at all (score– 0), several days (score– 1), more than half the days (score– 2), and nearly every day (score– 3) [33]. Scores of the questions range from 0 to 27, and are grouped for screening depression as follows: 0–4 points– minimal depression; 5–9 points– mild depression; 10–14 points– moderate depression; 15–19 points– moderately severe depression; 20–27 points– severe depression [31]. However, for convenience of analysis, we classified the scores into two categories: (i) no depression (minimal-mild depression, 0–9 points; code 0), and (ii) depression (moderate to severe depression, 10–27 points; code 1). In the PHQ-9, there is an additional question for determining severity of impairment of functioning that was not considered in this study as our focus was on gender-based analysis of the prevalence and predictors of depression among married adults.

### Independent variables

We considered some anthropometric, demographic, socio-economic, household, familial and health-related factors as independent variables for this study (Table 1).

### Statistical analysis

Frequency distribution was used to calculate the prevalence of depression. Both simple and multiple binary logistic models were used to find the association between selected factors and depression. All variables with a p-value of < 0.20 from the likelihood ratio test in the simple model were included in the multiple logistic regression model corresponding to each variable in the underlying multiple logistic regression model:

log [*P*/ (1 − *P*)] = β_0_ + β_1_* × *_1_ + β_2_* × *_2_ + β_3_* × *_3_ + β_4_*X*_4_ + β_5_*X*_5_ + β_6_*X*_6_ + β_7_*X*_7_ + β_8_*X*_8_ + β_9_*X*_9_ + β_10_*X*_10_ + β_11_*X*_11_ + β_12_*X*_12_, …………………………(1).

where, *P* = the probability of depression, 1 − *P* = the probability of no depression, *X*_1_ = nutritional status, *X*_2_ = age, *X*_3_ = relationship with spouse, *X*_4_ = number of marriages, *X*_5_ = number of family members, *X*_6_ = respondent’s education level, *X*_7_ = chronic medical comorbidity, *X*_8_ = age at the first marriage, *X*_9_ = family’s monthly income, *X*_10_ = number of children, *X*_11_ = respondent’s occupation, *X*_12_ = duration of conjugal life; β_0_ = intercept term, and β_i_ = unknown logistic regression coefficients (i = 1, 2, 3, …,12). The parameter β_i_ refers to the effect of *X*_i_ on the log odds such that *Y* = 1, controlling the other *X*_i_. The magnitude of the standard error (SE) was used to check the multicollinearity problem among independent variables; as the magnitude of the SE lay between 0.001 and 0.5, it was judged that no multicollinearity problem existed among the independent variables [36]. The statistical package for the social sciences (SPSS, version 23.0) was used for all the analysis. A 5% level of significance was considered.

## Results

A total of 708 married adults took part in the study. Males and females were of equal number (354). Their mean age was 33.21 ± 10.39 years (male 35.94 ± 10.41 years and female 30.48 ± 9.64 years). About 80% (male 73.4% and female 85.0%) of them were young adults. Nearly one-third (32.6%) (male 29.7% and female 33.9%) and only 6.8% (male 5.1% and female 6.2%) of the respondents were over- and under-nourished respectively, and 15.3% (male 10.2% and female 20.3%) got multiple marriages. More than 23.0% of the participants were living in poor family (monthly income ≤ Tk. 20,000), 41.0% had ≥ 5 family members, while only 6.2% had 2 members; and 87.85% had at least one live child. More than one-third (34.7%) of the respondents passed their conjugal life of 1–6 years followed by 7–12 years (33.1%) and ≥ 13 years (32.2%). 28.0% (male 16.9% and female 39.0%), 61.4% (male 66.9% and female 55.9%) and 10.6% (male 16.1% and female 5.1%) of them got their first marriage at < 18 years, 18–25 years and ≥ 26 years respectively. The literacy rate of the subjects was 89.41% (male 90.1% and female 88.7%), and 39.0% (male 34.2% and female 43.8%) had chronic medical comorbidity. 52.54% (male 46.9% and female 58.2%) of the respondents had poor relationship with their spouse; and most of the females were (91.8%) housewives, while 74.4% males were doing hard work (farmer or labor or others) (Table 1).

**Table 1:** General characteristics of samples

Variables	Frequency, N (%)	Variables	Frequency, N (%)
**Age** ≤40 years >40 years	561 (79.2) 147 (20.8)	**Nutritional status** Under-nourished (BMI < 18.5) Healthy (BMI 18.51–24.9) Over-nourished (BMI ≥ 25)	48 (6.8) 429 (60.6) 231 (32.6)
**Gender** Male Female	354 (50.0) 354 (50.0)	**Number of marriages** Single Multiple	600 (84.7) 108 (15.3)
**Family’s monthly income** ≤Tk. 20,000 >Tk. 20,000	164 (23.2) 544 (76.8)	**Age at the first marriage** <18 years 18–25 years ≥26 years	198 (28.0) 435 (61.4) 75 (10.6)
**Respondent’s education level** Illiteracy Literacy	75 (10.59) 633 (89.41)	**Chronic medical comorbidity** No Yes	432 (61.0) 276 (39.0)
**Number of live children** No child Having children	86 (12.15) 622 (87.85)	**Relationship with spouse** Poor Good	372 (52.54) 336 (47.46)
**Occupation of male** Service Hard work (Farmer or labor or others)	181 (25.6) 527 (74.4)	**Number of family members** 2 members 3–4 members ≥ 5 members	44 (6.2) 374 (52.8) 290 (41.0)
**Occupation of female** Housewife Others	91.8% 8.2%	**Duration of conjugal life** 1–6 years 7–12 years ≥ 13 years	246 (34.7) 234 (33.1) 228 (32.2)

**Fig. 1:**
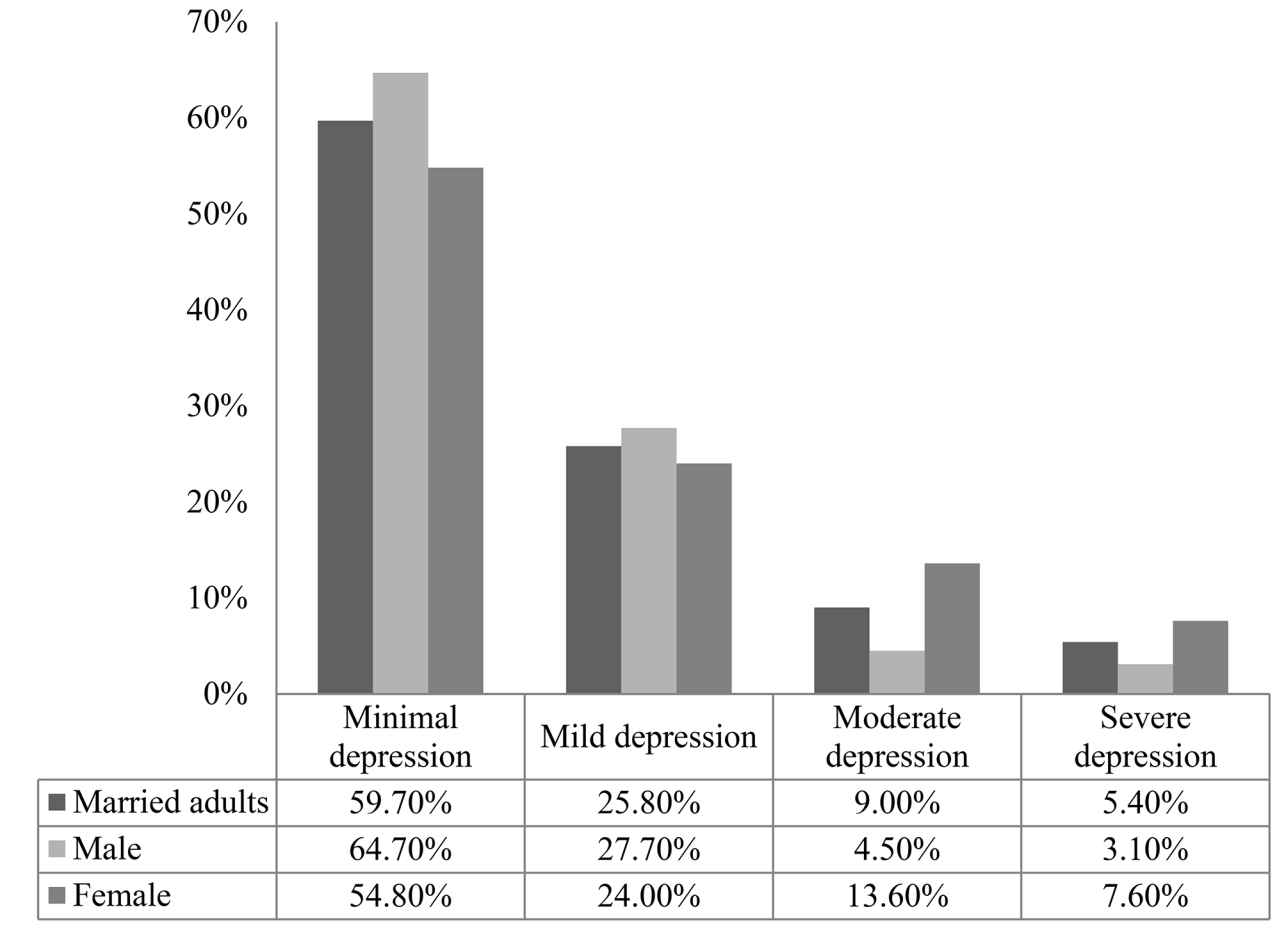
Levels of depression among married adults, male and female in Rajshahi city of Bangladesh

**Fig. 2:**
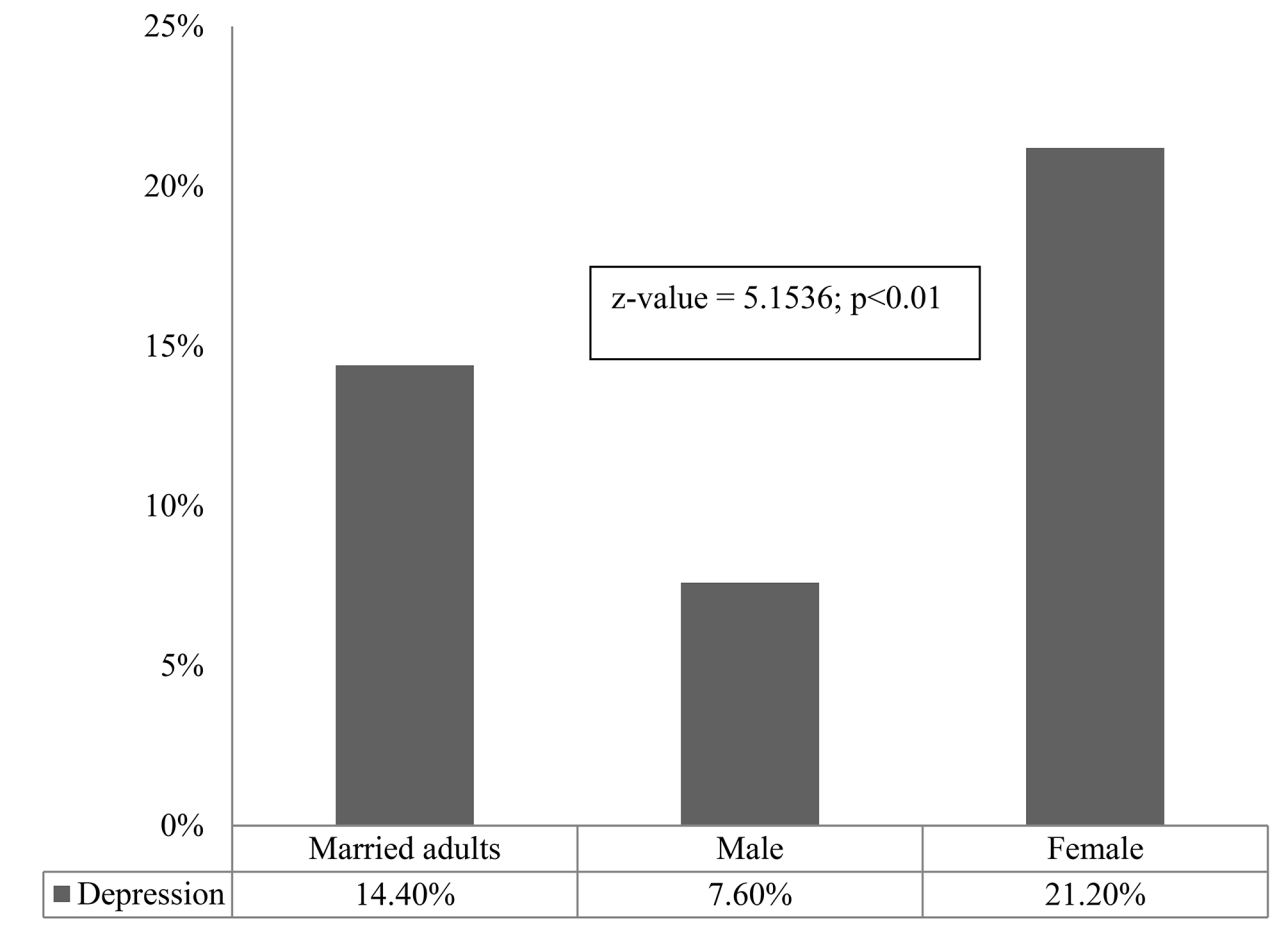
Prevalence of depression among married adults, and males and females in Rajshahi city of Bangladesh

It was found that 25.8%, 9.0% and 5.4% married adults were suffering from mild, moderate and severe depression respectively. Females had higher rate of moderate (13.6%) and severe (7.6%) depression than males (Fig. 1). The study revealed that 14.4% (95% CI: 11.9–16.9) of married adults were suffering from depression (Fig. 2). The prevalence of depression among females (21.2%; 95% CI: 17.2–25.4) was significantly (p < 0.01) higher than that of males (7.6%; 95% CI: 4.8–10.5) (Fig. 2).

It was observed that the highest number (31.8%) of undernourished females suffered from depression while only 5.6% of undernourished males were depressive. Younger adult females (≤ 40 years) had 22.6% and their counterpart males had 9.60% of depression. The prevalence of depression (27.7%) was more than four times higher among females who had poor relationship with spouse compared to their male counterparts (6.6%). More than seven times (68.1%) higher prevalence of depression was found among females compared to males (9.2%) who had multiple marriages. Of those who lived in small family (2 members), 27.30% of males and 31.8% of females suffered from depression. We found that illiterate subjects (25.0% of females and 20.0% of males) had a higher correlation to depression (Fig. 3a).

**Fig. 3a:**
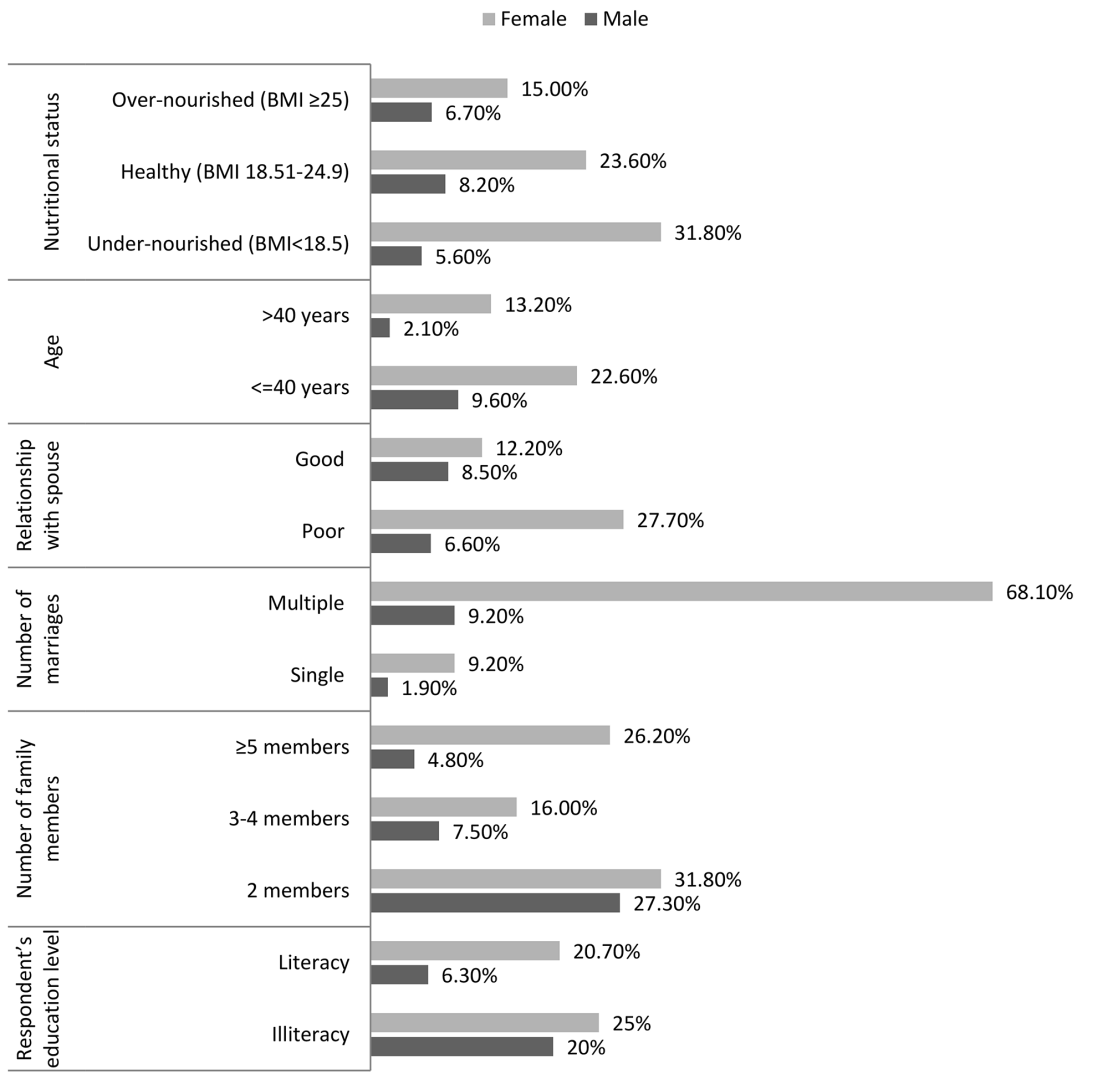
The prevalence of depression among males and females separately by their different characteristics

Males and females experiencing conjugal life of 7–12 years had the highest 11.1% and 27.4% prevalence of depression compared to others. More than one-fourth (27.70%) of females and 8.3% of males suffering from chronic medical comorbidity had depression. Increasing tendency of depression with increasing age at the first marriage was found among females. Females living in low income (≤ Tk.20,000) family had a higher rate (21.0%) of depression compared to males (8.6%). A higher number of males (18.60%) and females (25.60%) suffered from depression who did not have child compared to their counterparts, and hard-working males (10.3%) suffered from a higher rate of depression (Fig. 3b).

**Fig. 3b:**
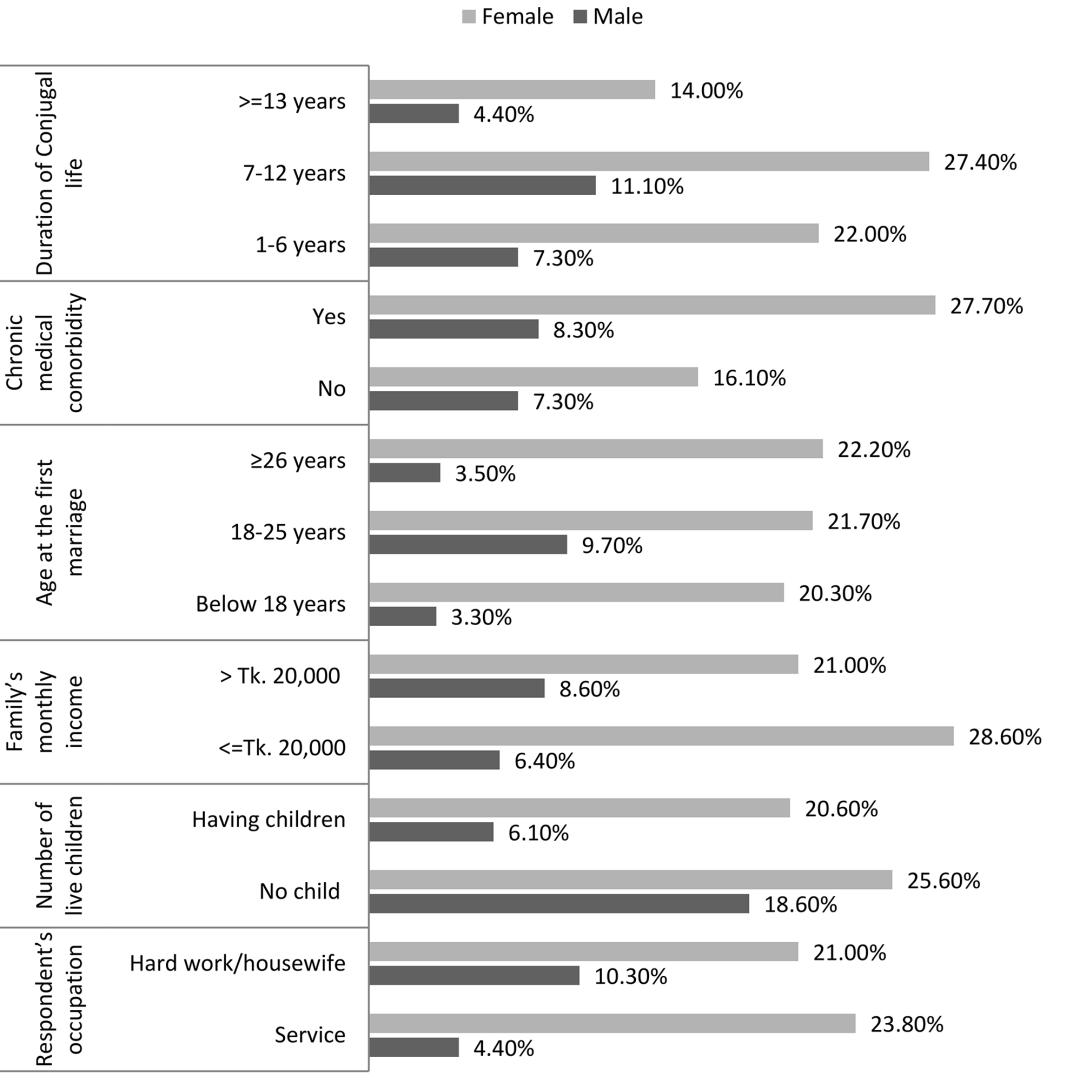
The prevalence of depression among males and females separately by their different characteristics

Table 2 shows the crude and adjusted odds ratio of depression by different background characteristics among males and females separately. We selected independent variables for multiple logistic model on the basis of p-value (p-value < 0.2) from simple logistic regression model. The factors were age, nutritional status, relationship with spouse, number of marriages, number of family members, respondent’s educational level, duration of conjugal life, chronic medical comorbidity, age at the first marriage, family’s monthly income, number of live children, and respondent’s occupation. The magnitude of the standard error (SE) showed that there was no evidence of multicollinearity problems among the independent variables. Multiple regression model demonstrated that number of marriages was an important predictor of depression for both males and females as compared to single marriage; multiple marriage showed 24.615- and 19.982-fold higher chance to develop depression among males [AOR = 24.605; 95% CI: 20.228–40.402; p < 0.01] and females [AOR = 19.982; 95% CI: 10.081–39.610; p < 0.01] respectively. Females who had poor relationship with their spouses showed a higher risk of depression [AOR = 2.175; 95%CI: 1.068–4.428; p < 0.05] than their counterparts. Females with chronic medical comorbidity [AOR = 1.876; 95%CI: 1.009–2.626; p < 0.05] was found 1.876 times more likely to have depression compared to those who did not have chronic medical comorbidity. Males doing hard work had more probability to get depression than males doing service [AOR = 4.358; 95%CI: 1.109–7.132; p < 0.05]. Females experiencing 7–12 years conjugal life were 2.091 times more likely to suffer from depression than females who already passed conjugal life of ≥ 13 years [AOR = 2.091, 1.009–4.334; p < 0.05].

**Table 2:** Impact of socio-economic, demographic and health related factors on depression of adult male and female

Variables		Male		Female	
Variable	Group	COR (95% CI: Lower-Upper; p-value)	AOR (95% CI: Lower-Upper; p-value)	COR (95% CI: Lower-Upper; p-value)	AOR (95% CI: Lower-Upper; p-value)
Nutritional status	Under nourished Vs Over nourished^R^	0.824 (0.095–7.124; p = 0.860)		2.644(0.946–7.389; p = 0.064)	1.150(0.321–4.120; p = 0.830)
	Healthy Vs Over nourished^R^	1.255(0.511–3.083; p = 0.621)		1.749(0.967–3.165; p = 0.065)	1.695(0.781–3.677; p = 0.182)
Age (years)	≤40 Vs > 40^R^	4.894(1.136–21.078; p = 0.033)	5.160(0.681–39.110; p = 0.112)	1.918(0.828–4.442; p = 0.129)	1.501(0.532–4.237; p = 0.443)
Relationship with spouse	Poor Vs Good^R^	0.763(0.344–1.694; p = 0.506)		2.763(1.547–4.933; p = 0.001)	2.175(1.068–4.428; p = 0.032)
Number of marriages	Multiple Vs Single^R^	22.800(19.609–42.955; p = 0.001)	24.605(20.228–40.402; p = 0.001)	20.977(11.073–39.737; p = 0.001)	19.982(10.081–39.610; p = 0.001)
Number of family members	2 members Vs ≥ 5 members^R^	7.393(2.211–24.719; p = 0.001)	1.709(0.167–17.527; p = 0.652)	1.314(0.498–3.468; p = 0.581)	1.183(0.306–4.577; p = 0.808)
	3–4 members Vs ≥ 5 members^R^	1.595(0.627–4.062; p = 0.327)	1.885(0.531–6.688; p = 0.326	0.538(0.314–0.922; p = 0.024)	0.947(0.472–1.902; p = 0.878)
Respondents’ education level	Illiteracy Vs Literacy^R^	3.737(1.454–9.605; p = 0.006)	1.815(0.292–11.276; p = 0.522)	0.277(0.594–2.747; p = 0.534)	
Chronic medical comorbidity	Yes Vs No^R^	1.145(0.507–2.583; p = 0.745)		2.004(1.195–3.358; p = 0.008)	1.876(1.009–2.626; p = 0.041)
Age at the first marriage (years)	18–25 Vs < 18^R^	3.117(0.714–13.607; p = 0.131)	2.723(0.411–18.044; p = 0.299)	1.090(0.638–1.861; p = 0.753)	
	≥ 26 Vs < 18^R^	1.055(0.144–7.748; p = 0.958)	3.369(0.260-43.704; p = 0.353)	1.122(0.343–3.675; p = 0.849)	
Family’s monthly income	≤Tk. 20,000 Vs > Tk.20,000^R^	0.720(0.320–1.621; p = 0.428)		1.501(0.285–7.896; p = 0.631)	
Number of children	No child Vs Having child^R^	3.513(1.432–8.617; p = 0.006)	2.747(0.453–16.672; p = 0.272)	1.327(0.634–2.775; p = 0.453)	
Respondent’s occupation	Hard work/housewife Vs Service^R^	2.512(1.034–6.104; p = 0.042)	4.358(1.109–7.132; p = 0.035)	0.852(0.302–2.405; p = 0.762)	
Duration of conjugal life	1–6 years Vs ≥ 13 years^R^	1.721(0.559–5.298; p = 0.344)	0.773 (0.219–2.724; p = 0.689)	1.723(0.873–3.398; p = 0.117)	1.544(0.725–3.284; p = 0.260)
	7–12 years Vs ≥ 13 years^R^	2.725(0.939–7.911; p = 0.065)	1.328(0.404–4.369; p = 0.641)	2.306(1.184–4.492; p = 0.014)	2.091(1.009–4.334; p = 0.047)
Hosmer and Lemeshow Test		Chi-square value = 5.683; p = 0.683	Nagelkerke R Square-value = 0.570	Chi-square value = 4.173; p = 0.841	Nagelkerke R Square-value = 0.432

** N. B.**: COR = Crude Odds Ratio; AOR = Adjusted Odds Ratio; CI = Confidence Interval; R = Reference

## Discussion

We designed this study aiming at determining the prevalence of depression among married adults in Rajshahi City of Bangladesh and examining its gender-based risk factors. For that purpose, we recruited 354 couples (708 married adults).

### Prevalence

We found that the prevalence of depression among married adults was high (14.4%). This finding could not be compared as no such previous study was available with this type of population. National Mental Health Survey (NMHS) 2019 reported that 6.7% of Bangladeshi adults (including both married and unmarried) were suffering from depressive disorder [17]. NMSH finding should be considered in the perspective that these cases were clinically diagnosed by psychiatrists. A recent study found that the prevalence of major depression was 17.9% among junior healthcare professionals of public teaching hospitals in Bangladesh [37]. It was revealed in a very recent study that 57.7% of undergraduate entrance admission seeking students in Bangladesh was suffering from mild to extremely severe depression [38]. There were some other studies that found 20.0–69.5% of different groups of populations in Bangladesh had depression [18–20, 24–27]. In some recent studies in abroad, the prevalence of depression varied remarkably. A study conducted in Uganda reported 27.7% prevalence of depression among adults [39]. A USA study found 8.1% of American adults had depression during 2013–2016 [40]. The prevalence of moderate, moderately severe and severe depression was recorded in Kerala of India at 4.6%, 2.5% and 0.9% respectively [41]. In a cohort study conducted from 2014 to 2017 in Iran, 5.2% of adults had depression [42]. In a systemic review and meta-analysis, an Ethiopian study recorded 41.85% prevalence of depression among older adults [43]. Another review study recorded the pooled prevalence of depression of 11.2% [44]. Compared to these reports, our finding might not be unjustified.

In this study, females were found to be at a higher risk of developing depression. The NMHS 2019 reported the same type of finding (male 5.4%, female 7.9%) [17]. Many other studies conducted with different groups of populations also supported our finding [12, 14, 15, 27].

### Risk factors

#### Multiple marriages

In the current study, multiple marriages was revealed as an important factor of depression in both males and females. This finding could not be compared as no study was found to discuss the effect of this factor on depression. Multiple marriage causes problems of divorce, absence of father/mother, step-parents/offspring/sibling, emotional distress, economic instability, jealousy, anger, poor family relationships, etc. that might contribute to develop many psychological problems like depression in both males and females.

#### Poor relationship with spouse

We found poor relationship with spouse as a contributory factor of depression among females. Poor family relationship has a negative impact on mental health [45], and is a risk factor of depression [46]. A systematic review reported that 58.2% of females and 46.9% of males felt they had poor relationship (self-perceived) with their spouses, and relationship problems and arguments among couples were positively correlated to depression and close confiding relationship was less likely to contribute to developing depression [15]. Due to the patriarchal family structure and social culture, females are to suffer most for relationship issues in Bangladesh, and that could explain the higher prevalence of depression among females in this current study.

#### Chronic medical comorbidity

Chronic medical comorbidity was found as an important predictor of depression among females. Some previous studies showed that chronic medical comorbidity had a significant impact on depression [13, 28, 29] to support our finding. This can be explained by the fact that, in our male-dominant family culture, any chronic disease makes the female partner lose her position and dignity in the family and make her a victim of negligence that might contribute to developing depression in her. This does not usually happen in the case of males.

#### 7–12 Years duration of conjugal life

Females who were passing 7–12 years of conjugal life comparatively suffered more from depression than their counterparts. This could not also be compared as no previous study considered this factor. In the early phase of conjugal life, couples are more romantic and they try to ignore all adversities and enjoy life. But, in the middle part of life, romance fades away. Now they have children and many other responsibilities. Hard realities continuously hurt them, and pains, sufferings, quarrels, harassments, disillusions become a part of their daily life. This might cause hopelessness and despair among them ultimately developing depression. However, in later life, couples are experienced enough about the realities of life that help them tolerate even bigger pains that help them to overcome depression. Duration of conjugal life did not significantly affect our male subjects, perhaps, because, in our patriarchal society, males are least sufferers, all sufferings usually and mostly go to the side of females making them more vulnerable than males.

#### Hard work

This factor was found to significantly affect depression among males. In two systematic review and meta-analytic studies, job-strains were reported to have significant correlation to depression [47, 48] that partly supports our finding. In Bangladesh, males are usually responsible for earning livelihood and managing other family issues. Hardworking people are poor, and they are to remain active and worried always with their responsibilities. They often burn out and get exhausted both physically and mentally. These strains might ultimately contribute to develop depression in them.

Though not statistically significant in multiple logistic regression analysis, number of family members showed a high correlation to depression in both genders. Respondents with two family members had the highest rate of depression in both males and females compared to other groups having more than two family members. As the participants were exclusively couples, we can assume that these subjects had no child. In Bangladesh, having no child is a source of chronic distress and social stigma for couples, especially for the female partners, and this might be a contributory factor of depression among them. In this study, illiteracy was found statistically correlated to depression among males, not in females. There are some studies in Bangladesh, India and Pakistan that reported similar findings [14, 15, 27]. In our families, most females, irrespective of their educational status, equally play role of homemaking and face almost the same types of problems. On the other hand, lack of education significantly decreases dominance, position and dignity of males in both the family and society that does not happen in the case of females to that much extent. That might be a big cause of hopelessness among males and a minor cause among females. In a male-dominant culture like Bangladesh, we know, sufferings from number of marriages, duration of conjugal life and relationship with spouse affects mostly the female gender. In addition, two other associated factors– homemaking and having no child– though not statistically significant in regression analysis, are mostly concerned with sufferings of females. All these factors are also family-related and inter-connected issues and thus corroborate one another in developing depression among couples, especially in female partners.

#### Strength of the study

It was a household study in which related issues were thoroughly and deeply searched. We focused on a new issue in this study and used appropriate statistical methods and models for sampling and data analysis and got significant findings. Two new associated factors (number of marriage and duration of conjugal life) were also identified.

#### Limitations of the study

There were some limitations of our study. Firstly, it was a cross-sectional study that could not establish causal relations. Thirdly, the PHQ-9 was not validated for the study population. Fourthly, some important factors such as family environment, traumatic event, history of depression among blood-connected relatives and treatment facility were not considered.

## Conclusions

The objective of this household cross-sectional study was to determine the prevalence of depression and examine its predictors among married adults in Rajshahi city of Bangladesh. We found some differences in the prevalence and risk factors of depression between males and females. The prevalence of depression among the respondents was significantly high, and the female gender showed a higher likelihood of developing depression. The predictors of depression were multiple marriages for both males and females, and poor relationship with spouse, chronic medical comorbidity and 7–12 years duration of conjugal life for females, and hard work for males. The study revealed that as a group of population, married females are the most vulnerable group for depression and family issues are strongly concerned. The government, non-government, social, cultural and religious organizations should take proper initiatives based on the findings of the study. Treatment and counseling facilities should be available and psychiatrists and psychologists should give special focus on family issues during treatment.

## Data Availability

The study was based on the primary data. The data are available from the corresponding author on reasonable request.

## Abbreviations

AOR: Adjusted Odd Ratio
CI: Confidence Interval
COR: Crude Odds Ratio
IBM: International Business Machines
SE: Standard error
SPSS: Statistical Package for the Social Sciences
